# Blended Digital and Face-to-Face Care for First-Episode Psychosis Treatment in Young People: Qualitative Study

**DOI:** 10.2196/18990

**Published:** 2020-07-28

**Authors:** Lee Valentine, Carla McEnery, Imogen Bell, Shaunagh O'Sullivan, Ingrid Pryor, John Gleeson, Sarah Bendall, Mario Alvarez-Jimenez

**Affiliations:** 1 Orygen Melbourne Australia; 2 Centre for Youth Mental Health University of Melbourne Melbourne Australia; 3 Centre for Mental Health Swinburne University of Technology Melbourne Australia; 4 School of Behavioural and Health Sciences Australian Catholic University Melbourne Australia; 5 Healthy Brain and Mind Research Centre Australian Catholic University Melbourne Australia

**Keywords:** Blended Treatment, Psychotic Disorders, Digital Intervention, Adolescent, Young Adults, mHealth

## Abstract

**Background:**

A small number of studies have found that digital mental health interventions can be feasible and acceptable for young people experiencing first-episode psychosis; however, little research has examined how they might be blended with face-to-face approaches in order to enhance care. Blended treatment refers to the integration of digital and face-to-face mental health care. It has the potential to capitalize on the evidence-based features of both individual modalities, while also exceeding the sum of its parts. This integration could bridge the online–offline treatment divide and better reflect the interconnected, and often complementary, ways young people navigate their everyday digital and physical lives.

**Objective:**

This study aimed to gain young people’s perspectives on the design and implementation of a blended model of care in first-episode psychosis treatment.

**Methods:**

This qualitative study was underpinned by an end-user development framework and was based on semistructured interviews with 10 participants aged 19 to 28 (mean 23.4, SD 2.62). A thematic analysis was used to analyze the data.

**Results:**

Three superordinate themes emerged relating to young people’s perspectives on the design and implementation of a blended model of care in first-episode psychosis treatment: (1) blended features, (2) cautions, and (3) therapeutic alliance.

**Conclusions:**

We found that young people were very enthusiastic about the prospect of blended models of mental health care, in so far as it was used to enhance their experience of traditional face-to-face treatment but not to replace it overall. Aspects of blended treatment that could enhance clinical care were readily identified by young people as increasing accessibility, continuity, and consolidation; accessing posttherapy support; strengthening the relationship between young person and clinician; and tracking personal data that could be used to better inform clinical decision making. Future research is needed to investigate the efficacy of blended models of care by evaluating its impact on the therapeutic alliance, clinical and social outcomes, cost-effectiveness, and engagement.

## Introduction

If you think about it, our online life is integrated with our physical life... It wouldn't really make sense not to try and integrate it.

Frieda, 23

The term *psychosis* refers to a serious mental health condition characterized by impairments in thought, perception, mood, and behavior and often manifests in hallucinations and delusions [1]. *First-episode psychosis* refers to the initial onset of psychosis and typically emerges in late adolescence and early adulthood [2,3]. Due to its onset during this important developmental stage, first-episode psychosis can cause severe disruptions to a young person’s education, employment, and interpersonal relationships [4]. If left untreated, first-episode psychosis can have grave long-term psychological and functional consequences and is often associated with persistent comorbid mental and physical health conditions [4].

Receiving treatment in the first 5 years following a diagnosis of psychosis may be critical to maximizing psychological and functional recovery [5-8]. Therefore, the development of early intervention services in the youth mental health field has been integral to improving clinical outcomes for young people experiencing first-episode psychosis [6,7].

While these improvements are significant in the short to medium term, there is evidence that positive clinical gains decline over time once a young person is discharged from early intervention services [9]. Current research suggests that providing treatment for a prolonged period of time (ie, extended across a full 5-year period following diagnosis) could be effective in maintaining clinical gains and improving social functioning over the longer term [7,10,11].

The term *digital intervention* refers to the digital delivery of psychosocial support and information, symptom monitoring, clinical connection, and peer connection through the use of technology such as computers, smartphones, and wearables [12]. Digital interventions have the potential to provide efficient avenues to extend treatment for young people experiencing first-episode psychosis [3,13,14]. It is estimated that up to 89% of people aged 16 to 25 years access social media daily [15], which makes digital intervention particularly relevant to adolescent and young adult populations. Importantly, young people experiencing first-episode psychosis also endorse digital pathways as a valuable mode through which to access mental health support and information [13,15,16]. To date, however, uptake of and engagement with digital mental health interventions are low, and attrition rates for mental health interventions, in general, are high [13,17]. Furthermore, most online interventions are focused on initial or short-term engagement, and little is known about maintaining long-term engagement [18].

Some studies have found that digital mental health interventions can be feasible and acceptable for young people experiencing first-episode psychosis; however, not many have examined how they might be blended with face-to-face approaches in order to enhance care [19]. The term *blended treatment* refers to the integration of digital and face-to-face mental health care [20], which has the potential to capitalize on the evidence-based features of both individual modalities while exceeding the sum of its parts; this integration could bridge the online–offline treatment divide and better reflect the interconnected, and often complementary, ways young people navigate their everyday digital and physical lives.

In this study, face-to-face treatment refers to the specialist service provided by the Early Psychosis Prevention & Intervention Centre which is delivered at Orygen, a mental health service for young people aged 15 to 25 years who reside in the western and northwestern regions of metropolitan Melbourne, Australia. Specifically, the Early Psychosis Prevention & Intervention Centre provides recovery-oriented care for a period of up to 2 years to young people experiencing first-episode psychosis [21]. The service includes weekly case management, medication management, carer support, and psychosocial groups [21]. Alternatively, a digital intervention refers to the centralized online platform, Horyzons [3], that affords young people digital access to evidence-based therapy, an online social network, clinical support, and peer support. A blended model of care in this study, therefore, refers to an interconnected model of digital and face-to-face treatment. In this context, a young person may have access 24 hours a day/7 days a week to a digital platform that delivers personalized evidence-based therapy, social connection with other young people, peer moderation, and clinical moderation delivered by their face-to-face clinician, in addition to their regular case management service.

Initial uptake and sustained adherence to digital mental health interventions remain low [13,17]. This is problematic as young people may not receive the minimum dosage of digital treatment necessary in order to receive positive clinical effects [22]. Involving young people in the development of mental health services and digital interventions has been recognized as both a practical and ethical imperative in the mental health domain [23]. Involving end users, the young people that the service is intended for, in the design and implementation phase increases both usability and credibility [17], which may lead to higher levels of engagement overall [17].

To date, little is known about young people’s perspectives on blended models of face-to-face and digital treatment in first-episode psychosis or in mental health treatment in general. Therefore, this study aimed to gain young people’s perspectives on the design and implementation of a blended model of care in first-episode psychosis treatment. 

## Methods

### Study Setting and Design

This qualitative study was underpinned by an end-user development framework [24] and based on semistructured interviews as part of a broader blended treatment research project at Orygen Digital, Orygen’s digital mental health arm. End-user development has traditionally been used in the human-computer interaction and software development fields to anticipate and flexibly address the needs of the intended user [24]. With the advent of digital intervention in the psychological field over the last decade, end-user development is now widely used in psychological research as an effective framework to gather and incorporate relevant feedback into the design and implementation of an intervention in a cost-effective and timely way [24].

**Figure 1:**
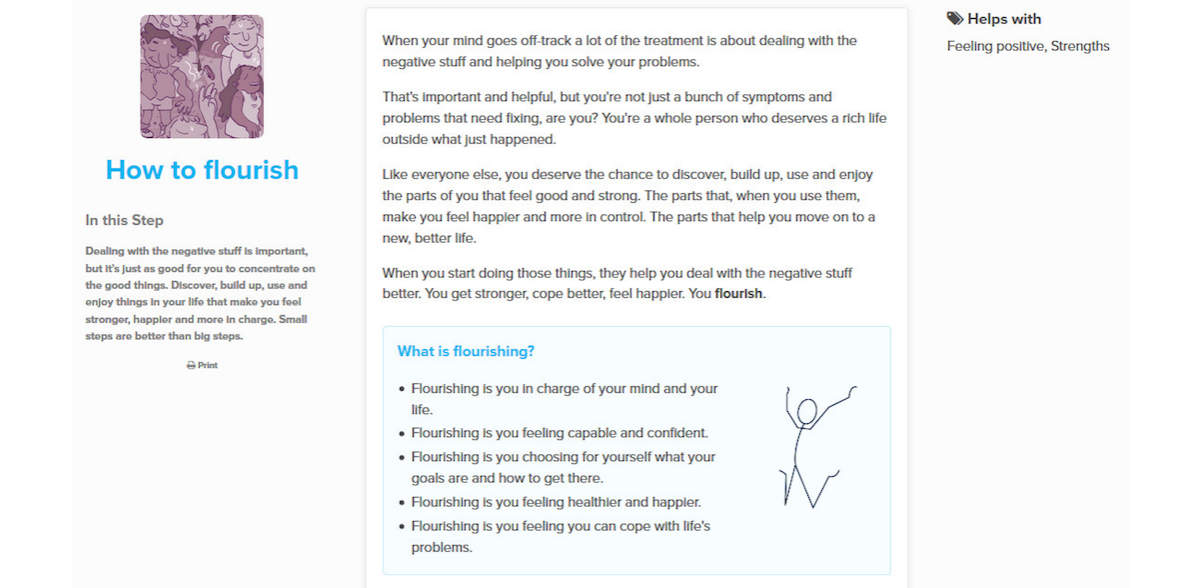
How to flourish webpage.

**Figure 2:**
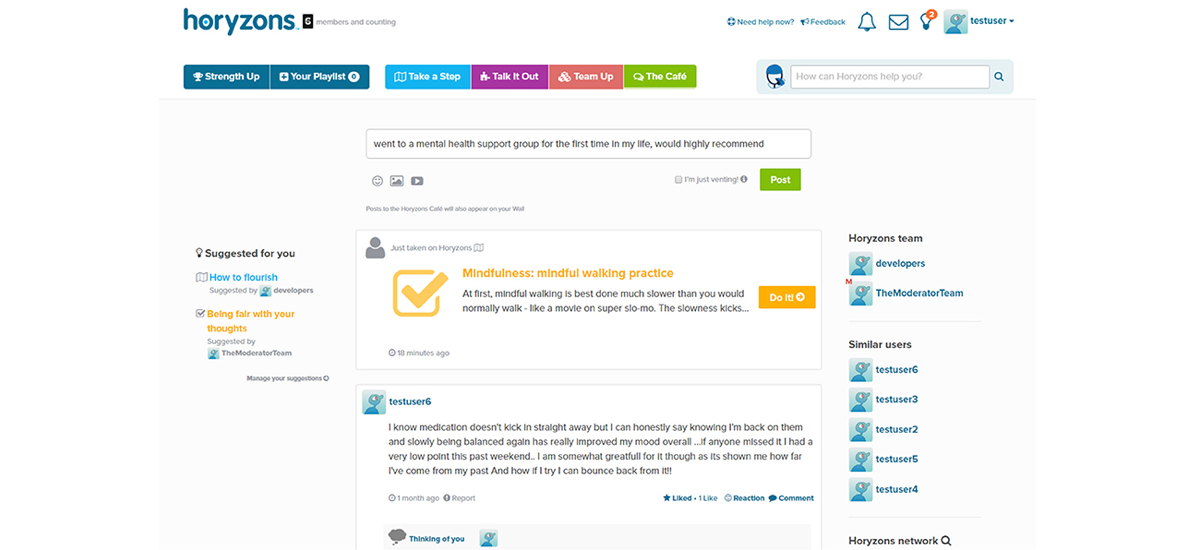
Interactive online social network webpage.

In this study, participants had the unique experience of completing a previous 18-month to 2-year face-to-face engagement period with the Early Psychosis Prevention & Intervention Centre, followed by participation in a long-term digital mental health intervention known as Horyzons. The Horyzons randomized controlled trial [3] randomly allocated participants, who were from 16 to 27 years of age, to either an intervention (n=85; access to the Horyzons platform in addition to treatment as usual) or a control condition (n=85; treatment as usual). The Horyzons platform was a multicomponent digital platform that included evidence-based online therapy (Figure 1), peer moderation, clinical moderation, and an interactive online social network (Figure 2). Young people assigned to the intervention were allocated a key moderator who supported the participant in completing relevant therapy modules, participating in group discussions, interacting on the social network, and engaging in individual web-based conversations with clinical and peer moderators. The platform is discussed in length in the Horyzons trial protocol paper [3]. The Horyzons randomized controlled trial was registered in the Australian New Zealand Clinical Trials Registry (ACTRN12614000009617).

### Participants

Young people were eligible to participate in this study if they (1) were previous clients of the Early Psychosis Prevention & Intervention Centre in Melbourne, Australia and (2) had been allocated to the intervention arm of the Horyzons randomized controlled trial.

Young people who met the criteria (n=10) were randomly contacted via phone call or text and invited to participate in a semistructured qualitative interview exploring their perspectives on the design and implementation of a blended model of face-to-face and digital mental health treatment. All 10 consented and were interviewed for the study.

### Data Collection

Ethics approval was obtained from the Melbourne Health Research and Ethics Committee in 2018 (HREC/13/MH/164). Data were collected from October 2018 to March 2019. Participants specified their preferred interview locations, and as such, interviews took place in a variety of locations which included the Orygen clinic, libraries, cafes, and participants’ homes. A semistructured interview guide was designed to explore participant perspectives of blended models of face-to-face and digital treatment for first-episode psychosis. The interview questions focused on perspectives on blended models of care, perspectives on how to deliver blended models of care, and perspectives on functions to include in a blended model of care. Author LV had previously established relationships with all of the participants as a research assistant on the Horyzons randomized controlled trial. Before the interview commenced, participants were provided with a plain language consent form and an information statement detailing the study, and they were given the opportunity to ask questions about the study. All interviews were audio-recorded and transcribed verbatim. All participants were reimbursed Aus $20 (approximately US $13.91) for their participation.

### Data Analysis

Given the end-user development framework and exploratory nature of the study, thematic analysis was considered the most appropriate method of data analysis [25]. Author LV completed the analysis and was supervised in this process by senior author SB. SB and LV had regular face-to-face meetings in which codes, themes, and LV’s thematic interpretations were interrogated thoroughly by SB to ensure rigor. Any disparity between authors regarding the analysis was debated until a resolution was reached. There were no a priori themes; all themes were derived directly from the data during the analysis process. Familiarization with the data was achieved by reading and rereading participant transcripts. Initial codes were assigned to the transcript to signify meaning, and codes that were similar within and between transcripts were noted. Aligned with a thematic analysis framework, the codes within and between transcripts were grouped into preliminary themes and reviewed in relation to all other themes. Some themes were superordinate—that is, the theme represented a whole category—while other themes were subordinate to the larger themes—these became subthemes [25]. In accordance with Morse’s [26] recommendations to maintain rigor, thick and rich descriptions of the themes were written up, these rich descriptions were then further debated between authors LV and SB.

## Results

### Overview

Participants (n=10) aged 19 to 28 (mean 23.4, SD 2.62) years were included in the study; 70% (7/10) were female, and 30% (3/10) were male. Female participants were all cisgender. Of the male participants, 2 were cisgender and 1 was transgender (Table 1).

**Figure 3:**
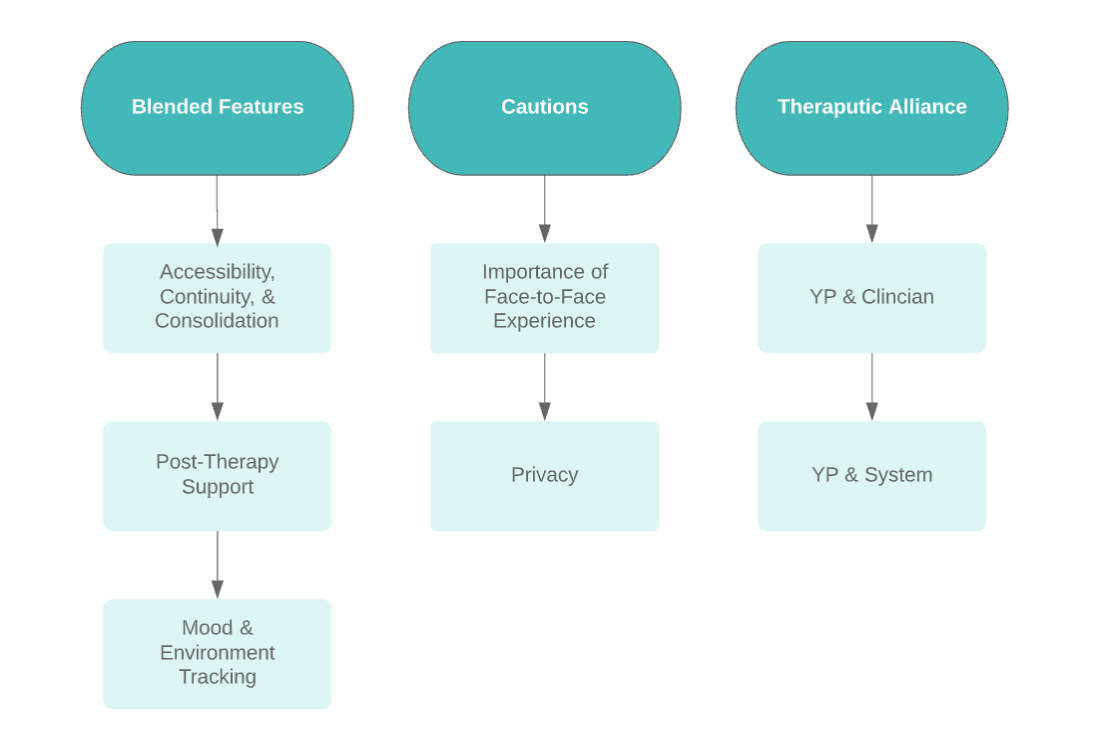
Superordinate and subordinate themes.

Three themes emerged relating to young people’s perspectives on the design and implementation of a blended model of care in first-episode psychosis treatment: (1) blended features, (2) cautions, and (3) therapeutic alliance (Figure 3).

**Table 1:** Alias, age, and gender of participants.

Alias	Age	Gender
Tristan	25	Male
Justine	19	Female
Ling	25	Female
George	22	Male
Amita	22	Female
Caroline	26	Female
Milly	24	Female
Frieda	23	Female
Vinh	28	Male
Isla	20	Female

### Blended Features

#### Accessibility, Continuity, and Consolidation

Having multiple pathways to a case manager was identified by almost all participants as an important accessibility feature of a blended model of treatment. For instance, Tristan identified that access to his therapist through a digital platform would be useful when it was not “realistic” to get to a physical location or speak on the phone because of commitments such as travel or work. Similarly, Justine suggested that it would be beneficial to have access to her clinician through an online platform for those occasions when she could not attend her therapy appointment in person because

...maybe your mum can't drive you there or you can't take public transport ‘cause you're not in the mindset.

Justine, 19

In both Tristan and Justine’s scenarios, an alternate pathway to their clinician through a digital platform would safeguard against missed therapy sessions due to physical or psychological barriers.

Ling proposed that a digital platform could assist young people in moments when they are without a scheduled appointment with their clinician, but are in need of support. She suggested that, if faced with this common issue, young people could access resources and support via the platform instead. George reiterated that having resources and information available on a digital mental health platform would be useful because

...[it provides] people with quality information, rather than what they can possibly source themselves…so I guess it saves a lot of wasted time for that person, at least you’re getting curated knowledge from a bunch of professionals rather than just what Google tells you.

George, 22

Both Ling and Justine also noted that, in instances where young people were not able to speak with their clinician, they could connect with other young people online for peer support:

...there's many users that have been through what [you have] been through or are like going through the same thing, and [you] can talk to each other and connect to each other.

Ling, 25

Tristan also observed that a digital therapeutic platform could be helpful for young people who were linked in with a mental health service, but were on the waitlist to see a clinician because

...sometimes you got to wait weeks to see a psychologist.

Tristan, 25

He suggested that the platform could offer assistance during this time period because

...it's accessible and it's completely, like, anonymous. So, like, you have that information there in a time of need.

Tristan, 25

Finally, he noted that there are times when young people can feel overwhelmed by the amount of therapeutic information or by the content of a therapy session. He suggested that a digital platform could be used to help consolidate the information, for instance,

...instead of walking away and potentially forgetting half of that information, you can kind of follow-up [on the platform] and kind of refresh or maybe pull apart some of the things that you were unable to in that session.

Tristan, 25

#### Posttherapy Support

Multiple participants acknowledged feeling sad or “down” after a therapy session and identified that the platform could be used in the postsession period to combat negative feelings and feel connected to others on the therapeutic social network who may be having similar experiences. For instance,

...sometimes after a session, you talk about all your feelings and then you just have to go back to your life. It would be good to have an app in those moments, something to go back to...

Amita, 22

#### Mood and Environment Tracking

The majority of participants identified that a digital function for tracking mood and environment could be useful in blended mental health treatment. One participant, however, noted that young people may purposively underreport mood.

Amita identified that mood tracking apps had been useful to her in the past and proposed that pertinent measures could be identified in collaboration with her clinician, and the digital platform configured to track relevant data and make personalized suggestions based on her information. For example,

Hey, you’ve been feeling really irritable and hot, and you’re getting headaches. Maybe you’re dehydrated. Drink some water.

Amita, 22

Caroline suggested that the ability to record the environment in addition to her mood would be helpful as she has noticed that her environment could have an impact on her mood and levels of paranoia. For instance,

I feel really suffocated here ‘cause I don't like being around people or like be in this type of situation. But, if I'm in an open space I feel more isolated but relaxed and calm, and I don't feel paranoid as much.

Caroline, 26

Caroline identified that if she could reflect on her user data with her clinician, she could make informed decisions about her environmental choices based on this information.

Alongside Caroline’s suggestion to record the environment as well as mood, George suggested that there could also be an option to record hashtags or keywords that are relevant to the young person’s thoughts and feelings. He noted that being made aware of correlations between particular mood trends and particular hashtags in order to better guide positive decision making could be an incentive to use the digital platform. Furthermore, he suggested that all user hashtags could be collated anonymously on the digital platform to generate a public cloud that could act to normalize young people’s thoughts and feelings to the broader group and be used to inspire conversational topics on the social network or in therapy sessions.

Milly advised that, in the early stages of her mental health care, she would underreport the depth of her low moods as she was afraid her treating team might increase her medication as a result. She said,

I didn't want them to know that I was really down, 'cause I didn't want my meds to increase. That was the main thing. I didn't like taking meds.

Milly, 24

She identified that other young people might also underreport the extent of their symptoms on a mood or mental health tracker for similar reasons.

### Cautions

#### Importance of Face-to-Face Contact

While participants expressed strong enthusiasm for a blended model of care, most were careful to communicate that they did not want a digital platform to replace the physical face-to-face experience of mental health treatment.

Tristan identified that he took information that was delivered to him face-to-face from a clinician more seriously than he took online advice because of the impact of the clinician’s tone of voice and body language. For Tristan, a digital platform could not embody these aspects of communication, and he acknowledged that if his Horyzons moderator had not, by complete chance, also been his face-to-face clinician then he may not have taken her digital therapy suggestions “as seriously” as he did. In a similar vein, both Isla and Ling shared that it can be harder to talk online and find the right words to express yourself, but it can be easier in person because “emotions just show on your face.” Isla also commented that purely online conversation could lead to miscommunication that would not be conducive to the therapeutic relationship, for instance, she said,

...like “hi” it can be processed in a polite way, and it can be processed in a rude way or [an] ignorant way, it really depends on what you see really.

Isla, 20

Vinh and Justine suggested it can be challenging for young people to discuss new or sensitive information in person and identified that the young person could speak to their clinician via webchat, even in the same room, as a way to work up to speaking about the topic face-to-face.

#### Privacy

Amita acknowledged that young people are not always open and honest with their clinicians and suggested they may be less inclined to share openly on a digital platform if they knew it would be read directly by their treating clinicians. Similarly, Caroline asserted that she would no longer share openly on a digital platform if she was aware her clinician could see what she was posting and potentially change her course of treatment based on that information, particularly if it could result in a medication increase:

Yeah, maybe I wouldn't be too open [long pause] now that I think about it yeah, I fully wouldn't.

Caroline, 26

### Therapeutic Alliance

#### Young Person and Clinician

Both George and Tristan were two participants interviewed who were randomly allocated an online moderator for the Horyzons randomized controlled trial that by chance also aligned with their face-to-face clinician at the Early Psychosis Prevention & Intervention Centre. They both felt that their relationships with their clinicians developed at a faster rate because they were “exposed” to each other more—both online and face-to-face. Tristan expressed that he only completed tasks on the Horyzons platform because they were set for him by his face-to-face clinician. He believed, “you could pull away more easily” from a moderator if you had not previously met them face-to-face. Tristan observed that it would be “a game-changer” for young people’s mental health treatment if the moderator were also the young person’s face-to-face clinician.

Tristan identified that having work set on the app between face-to-face therapy sessions would encourage him to complete it during the week due to a sense of accountability to his clinician, for instance,

Oh, I'm going to see her next week. Should probably get that done before she mentions it in my session.

Tristan, 25

Ling identified “regularity” as a key factor in increasing her comfort levels with a clinician and identified increased time spent in a digital and face-to-face combination could contribute to this experience. George and Milly both advised that having digital access to their clinician outside of a face-to-face session through a digital platform could “strengthen” the relationship. Similarly, Justine suggested that a digital platform used in conjunction with face-to-face treatment would be a means to feel connected and supported by your clinician “even when they’re not there.”

#### Young Person and System

Amita suggested a chatbot could be useful in fulfilling therapeutic needs. Chatbots have been identified as effective and enjoyable emerging technology within the mental health landscape [27]. The digital tool refers to hardware or software that uses artificial intelligence to imitate human dialogue in a task-orientated interaction between itself and a person [27]. Amita identified that she would like someone to talk to “...24 [hours a day],” and she “literally, wouldn’t mind” if that need was serviced through a chatbot: 

Like, sometimes people just have to say blah, blah, blah, blah, blah and then they feel fine… Like, sometimes I literally just have to scream on the keyboard.

Amita, 22

Furthermore, if the chatbot was an automated function, Amita noted that it would require less “effort” for her to speak to it than to a human clinician.

## Discussion

### Principal Findings

This qualitative study used an end-user design approach to explore young people’s perspectives on blended models of face-to-face and digital care in first-episode psychosis treatment. We found that perspectives could be grouped into three overarching themes and that these themes can be practically applied to the design and implementation of emerging blended models of mental health care.

We found that young people were very enthusiastic about the prospect of blended models of mental health care, in so far as it was used to enhance their experience of traditional face-to-face treatment but not to replace it overall. Aspects of blended treatment that could enhance clinical care were readily identified by young people as increasing accessibility, continuity, and consolidation; accessing posttherapy support; strengthening the relationship between young person and clinician; and tracking personal data that could be used to better inform clinical decision making. Overall, young people communicated that the digital experience could not embody the communication characteristics of face-to-face treatment. Tone, body language, and facial expressions were identified as important aspects of communication that young people felt would be lacking in a purely digital space. Previous research [28], however, has suggested that the benefit of online anonymity is particularly beneficial to some participant groups, and this benefit can work to counterbalance the loss of nonverbal communication. Furthermore, the potential that young people may underreport symptoms on mood tracking tools was identified. Concern centered on fears of clinicians and services responding to changes in clinical states in unwanted ways, such as changes to medications when reporting low mood. This has been reported in other studies [29,30], reflecting the importance of considering the role of, and response to, personal clinical information collected in daily life from users of clinical services.

Tracking mood, together with the environment and related keywords, was identified as a useful tool to guide positive decision making. Ecological momentary assessment is a method of recording momentary experiences using a mobile device in the context of daily life [31]. There has been recent interest in the use of information collected in daily life for clinical purposes. For example, one research group [32,33] explored the integration of ecological momentary assessment with standard psychological therapy for people with voice-hearing experiences. People tracked their voice-hearing experiences (and the context in which they occurred) over a week, allowing a clinician to analyze the patterns of occurrence. This was used to provide a formulation of the voices, which informed the selection of relevant intervention strategies. In our study, the use of tracking tools or ecological momentary assessment to identify mood patterns that could be subsequently shared with a clinician to the benefit of the young person's therapy was identified as potentially useful. The incorporation of a digital tracking tool could support young people working collaboratively with their clinicians in identifying patterns of symptoms, environments, and relationships with other determinants to inform treatment decisions and enable momentary interventions. This form of collaboration between young people and clinicians is aligned with autonomous and strength-based practice [34]. Importantly, in line with themes from our study, findings of qualitative research have suggested that this approach may fast-track treatment processes by enhancing the personalization of therapy, which would enable more individualized and targeted intervention strategies to be produced more efficiently based on the information gathered [35]. Additionally, an ongoing connection with a clinician, who has access to personal insights about the young person through their data, has the potential to enhance the therapeutic alliance.

The therapeutic alliance refers to the goal-directed, collaborative relationship between client and clinician and is the strongest predictor of client mental health outcomes [36,37]. According to Bordin [38], the alliance comprises three core conceptual elements which include bond, tasks, and goals [36]. One of the strongest findings identified in this study was the perception that a blended model of treatment had the potential to enhance the therapeutic relationship. Integrating a digital platform with the face-to-face experience was consistently endorsed as a means to strengthen the relationship between the young person and their clinician by providing greater consistency (ie, accessible sessions and more regular contact), collaboration (ie, joint decision making), accountability (ie, a sense of commitment to complete tasks on time), and the opportunity to consolidate information. This supports previous findings from Lederman et al [37], in which the experience of technology-mediated mental health therapy was examined and that, like Horyzons, included therapy pathways, a social network, clinical moderation, and peer moderation; Meridian was designed to support carers of young people experiencing mental illness. Lederman et al [37] found that the digital system could create an alliance-like experience for the users. Their research identified that it was likely that the combination of peer support workers, clinical moderators, and different core functions of the platform came together to “facilitate the formation of a state that mirrors therapeutic alliance [37].” The finding that a therapeutic relationship is possible beyond that of only the client–clinician experience is valuable knowledge to the digital mental health field, particularly because the therapeutic alliance is the strongest predictor of mental health outcomes [39].

In addition to the emerging possibility that the therapeutic alliance can develop beyond that of only the client–clinician relationship to incorporate a blended system (resembling client–technology–clinician), the notion of a digital therapeutic alliance between the user and the technology itself (ie, client–technology) is also a burgeoning area of research in the mental health field [27,37,40]. Among other technologies, chatbots are an avenue of such interest. Vaidyam et al [27] identified chatbots as an “effective” and “enjoyable” technology in the mental health setting. Chatbots were similarly acknowledged in this study, as a helpful venting tool and a plausible solution for a mental health service to provide sustainable 24-hour access. The concept of a digital therapeutic alliance between young people and technology or the digital platform itself is a notable avenue for further research.

For many young people who are native users of digital technology within their everyday lives [15] and are interested in receiving care via blended modalities, there is an important opportunity to assist them in strengthening connections with their therapist and offering ways to improve engagement with therapies. As the therapeutic alliance is considered one of the most important predictors of treatment efficacy regardless of the therapeutic approach [39], there is great potential in capitalizing on the ways to enhance this alliance through digital technologies or with digital technologies themselves [40]. The therapeutic alliance that develops through blended treatment has the potential to enhance engagement with both face-to-face and digital therapies through increasing therapeutic intensity and personalization, and by simultaneously addressing poor engagement and treatment continuity which are key issues in the digital health field [13,17].

### Future Research

Future research is needed to investigate the efficacy of blended models of care by evaluating its impact on the therapeutic alliance, engagement, and treatment effects. Additionally, the formation of a digital therapeutic alliance between a user and a digital platform or other technology is an emerging area of research worthy of further exploration.

### Limitations

Author LV had established relationships with all participants due to a prior role as a research assistant on the Horyzons randomized controlled trial and also completed all interviews in this study. While we found that the existing relationship could be conceptualized as a strength that possibly aided in creating a more open dialogue between interviewer and interviewees, a previous relationship generates an opportunity for participant bias as interviewees may, both consciously and unconsciously, provide answers that they believe will please the interviewer as opposed to what they may genuinely feel [41].

### Conclusions

Young people were very enthusiastic about the prospect of blended models in first-episode psychosis mental health treatment. Aspects of blended treatment that could enhance clinical care were readily identified by young people as increasing accessibility, continuity, and consolidation; accessing posttherapy support; strengthening the relationship between young person and clinician; and tracking personal data that could be used to better inform clinical decision making. Tone, body language, and facial expressions, however, were identified as important aspects of communication that young people felt may be lacking in a purely digital space. Furthermore, the potential of underreporting symptoms through mood tracking was identified. Blended care was identified as an avenue through which the experience of mental health treatment could be enhanced. Future research is needed to investigate the efficacy of blended models of care by evaluating its impact on the therapeutic alliance, clinical and social outcomes, cost-effectiveness, and engagement.
